# Phytochemical Characterization, Antioxidant, and Anti-Proliferative Activities of Wild and Cultivated *Nigella damascena* Species Collected in Sicily (Italy)

**DOI:** 10.3390/antiox13040402

**Published:** 2024-03-27

**Authors:** Monica Scognamiglio, Viviana Maresca, Adriana Basile, Severina Pacifico, Antonio Fiorentino, Maurizio Bruno, Natale Badalamenti, Marta Kapelusz, Pasquale Marino, Lucia Capasso, Paola Bontempo, Giuseppe Bazan

**Affiliations:** 1 Department of Environmental, Biological and Pharmaceutical Sciences and Technologies (DiSTABiF), University of Campania “Luigi Vanvitelli”, Via Vivaldi 43, 81100 Caserta, Italy; monica.scognamiglio@unicampania.it (M.S.); severina.pacifico@unicampania.it (S.P.); antonio.fiorentino@unicampania.it (A.F.); 2 Department of Biology, University of Naples “Federico II”, 80138 Naples, Italy; viviana.maresca@unina.it; 3 Department of Biological, Chemical and Pharmaceutical Sciences and Technologies (STEBICEF), University of Palermo, Viale delle Scienze, Ed. 17, 90128 Palermo, Italy; maurizio.bruno@unipa.it (M.B.); giuseppe.bazan@unipa.it (G.B.); 4 NBFC, National Biodiversity Future Center, 90133 Palermo, Italy; 5 Centro Interdipartimentale di Ricerca “Riutilizzo Bio-Based Degli Scarti da Matrici Agroalimentari” (RIVIVE), University of Palermo, 90128 Palermo, Italy; 6 Department of Biology and Pharmaceutical Botany, Medical University of Gdańsk, 80210 Gdańsk, Poland; martakap@gumed.edu.pl; 7 Bona Furtuna LLC, Los Gatos, CA 95030, USA; 8 Department of Precision Medicine, University of Campania “Luigi Vanvitelli”, Via L. De Crecchio 7, 80138 Naples, Italy; lucia.capasso2@unicampania.it (L.C.); paola.bontempo@unicampania.it (P.B.)

**Keywords:** antioxidant activity, antiproliferative activity, *Nigella damascena*, NMR, Ranunculaceae, triterpene saponins

## Abstract

The use of *Nigella damascena* seeds in the culinary field or as aerial parts infusions in the pharmaceutical and cosmetic fields is widely reported. The biological activity of this plant, as demonstrated over the years, is closely related to its phytochemical content. This investigation focused on the comparative study of the same plants of *N. damascena*, one totally wild (**WND**), while the other two, one with white flowers (**CWND**) and the other with blue flowers (**CBND**), were subject to cultivation, irrigation, and manual weeding. Using the potential of 1D and 2D-NMR spectroscopy, coupled with MS/MS spectrometric studies, the three methanolic extracts of *N. damascena* were investigated. Chemical studies have highlighted the presence of triterpene saponin compounds and various glycosylated flavonoids. Finally, the in vitro antiproliferative and antioxidant activities of the three individual extracts were evaluated. The antiproliferative activity performed on U-937, HL-60, and MCF-7 tumor cell lines highlighted a greater anticancer effect of the **CBND** and **CWND** extracts compared to the data obtained using **WND**. The antioxidant activity, however, performed to quantify ROS generation is comparable among the extracts used.

## 1. Introduction

**Figure 1:**
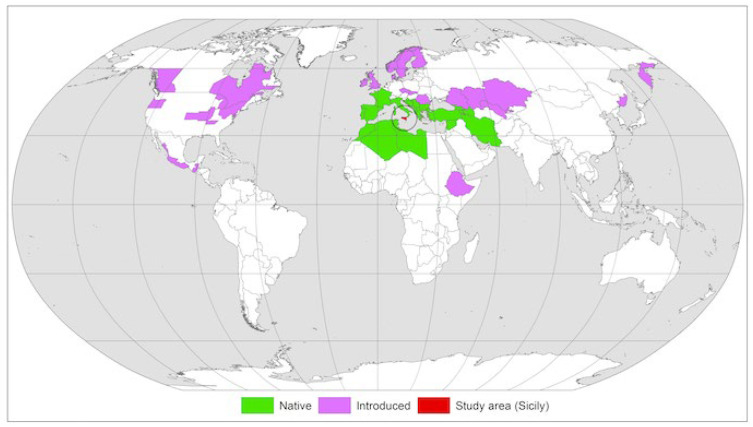
Global distribution of *N. damascena*. The black circle highlights the study area at the center of the natural distribution range [1,2].

*Nigella damascena* L., commonly *love-in-a-mist*, is a species with a Euro-Mediteranean distribution (Figure 1), growing spontaneously from Macaronesia to the Mediterranean basin (excluding Egypt and Israel) up to northwestern Iran [1].

**Figure 2:**
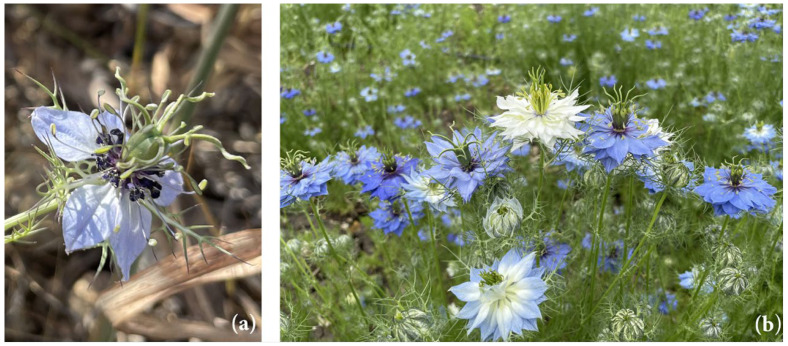
(**a**) The *N. damascena* flower, in which dark petals (reduced to nectaries) and white petaloids are evident, lends the species its ancient name, *Melanthium*, derived from the Greek *μέλαν* (melan) meaning “black” and *άνθος* (anthos) meaning “flower”. (**b**) In cultivated forms of *N. damascena* “Oxford Blue”, the flowers are characterized by having multiple verticils of petaloids ranging from white to blue during the development phase, which gives the species a high aesthetic value. The photos refer to the wild populations (**a**) and cultivated populations (**b**) sampled in Corleone for this study.

This species has a single, erect stem, which may branch at the top. The basal leaves are linear–spatulate, and the flowers are terminal, solitary, and surrounded by an involucre of filiform bractiform leaves (Figure 2a). The fruit of *N. damascena* are ovoid capsules, smooth and ribbed. When ripe, the fruit opens at the top to release numerous black, triangular, wrinkled, and aromatic seeds [3].

Commercially, *N. damascena* continues to be used in herbal medicine, with its seeds and derived oil being marketed as “black cumin”, a term encompassing both *N. damascena* and *N. sativa*. The primary interest in *N. damascena* today is as an ornamental garden plant and for its flowering beauty [4]. It is extensively cultivated in gardens for the aesthetic appeal of its numerous hybrid varieties [5], which feature double petaloid flowers in colors ranging from white to pink and various shades from blue to purple (Figure 2b) [6].

In its natural distribution range, this plant grows spontaneously in anthropogenic ecosystems: in fact, it tends to colonize disturbed areas, such as dry uncultivated fields, vineyards, arid fields and meadows, and on-the-road margins, from sea level to 1000 m a.s.l. [7].

This species is one of the 26 accepted taxa belonging to the genus *Nigella*, described by Linnaeus in *Species plantarum* [8] as one of the smaller genera of the Ranunculaceae family [4], which includes the better-known *Nigella sativa* (black cumin), a species widely consumed as a seasoning in North Africa, the Arabian Peninsula, and India.

Thus, if for *N. sativa,* the ancient medico-botanical literature of the past gives extensive treatment, *N. damascena* is considered of lesser interest [9]. The seeds of *N. damascena* L. are used as ingredients in a wide range of foods [10], for example, as flavorings in bread and cheese [11], and are widely present in the folk medicine of various countries and in traditional medicine, *N. damascena* seeds have been employed for menstrual regulation, indicating possible estrogenic effects [12], for catarrhal conditions, [13], for their pain-relieving, anti-edema, fever-reducing, worm-expelling, and disinfectant properties [14]. In Sicilian folk medicine, a seed infusion, referred to as *vaccareddi* or *lampiuneddi*, is used as a milk-production stimulant [15], but several cases of intoxication have been recorded [16].

The phytochemical analyses carried out on the extracts obtained from the different parts of *N. damascena* allowed us to distinguish two main classes of predominant metabolites, the alkaloids and the terpene derivatives. Carrying out an exhaustive analysis of the research on *N. damascena*, to date, 57 compounds have been isolated, and it is essential among these to number the various dolabbelanic diterpenes, complex structures often esterified with phenylacetyl, benzoyl, acetyl, nicotinoyl, and isobutyryl portions [3]. It should be noted that dolabellans are mostly products of marine organisms, such as mollusks or several algae [17], and that these are present in a few terrestrial plants [18,19,20], including *N. damascena* [21,22]. Over the years, different activities have been evaluated for this compound and among these, antimicrobial, anti-inflammatory [23], and anti-tumor [24] activities have been carried out.

The intention of this work aims to evaluate a qualitative diversity of polar extracts obtained from cultivated samples of *N. damascena* “Oxford Blue” (**CBND**), cultivated samples of *N. damascena* (**CWND**), and spontaneous plants collected from the wild (**WND**). Specifically, using 1D and 2D NMR techniques, coupled with UHPLC-ESI-QqTOF HR MS/MS experiments, the possible chemical diversity in qualitative terms on flavonoid, dolabellane, and triterpene compounds was evaluated. Subsequently, antioxidant and anticancer tests were carried out. The tests performed did not show the promising activity of the plants studied here, but they highlighted how phytochemical differences play a fundamental role in biological activity.

## 2. Materials and Methods

### 2.1. Plant Materials

The plant material consists of plants *N. damascena*, both wild and cultivated, at the full flowering stage. The samples were collected in May 2022 within the Bona Furtuna farm (Corleone, Italy), which funded the “Extremophytes Potential” project. This project aims to understand how extreme environmental conditions affect the production of biologically active compounds and how cultivation influences the synthesis of bioactive compounds that plants produce under stressful conditions. The collection site represents a significant example of the traditional rural landscape, characterized by a high degree of naturalness comprising elements of considerable naturalistic and environmental value.

The wild samples were taken from the natural population of *N. damascena* (**WND**) (37°45′49″ N 13°18′34″ E) located at an altitude of 770 m above sea level, growing in the Mediterranean dry grassland that settles at the base of Monte Barraù, a Special Conservation Zone (ZCS) of the Monti Sicani district.

The cultivated samples were taken from a cultivation plot within the Bona Furtuna farm (37°45′49″ N 13°18′23″ E) at an altitude of 760 m above sea level, about 300 m away from the natural population. These were the *N. damascena* “Oxford Blue” (**CBND**) and *N. damascena* (**CWND**), cultivated using traditional low-intensity methods to bring the plant to normal conditions of stress induced by the Mediterranean climate.

### 2.2. Extraction Procedure

For each sample (**WND, CWND,** and **CBND**), flowers, leaves, and stems, weighed using a technical scale (~88 g), once frozen (−20 °C), were subjected to a freeze-drying process until they reached a constant weight. Therefore, the freeze-dried plant material from each group (~20 g) was finely ground and then subjected to an extraction process at room temperature using methanol (50 mL × 3 times) (Sigma-Aldrich Corporation, St. Louis, MO, USA). After extraction procedures, the extracts were filtered, reduced in volume at low pressures and at low temperatures, and freeze-dried again until a constant weight was reached. The final yield was 27.95% for **WND**, 20.30% for **CWND**, and 19.96% for **CBND**.

### 2.3. Metabolite Profiling

Dried and powdered plant material (50 mg) for each sample type (**WND, CWND,** and **CBND**) was transferred to a 2 mL microtube and suspended in a mixture of 1.5 mL of phosphate buffer (Fluka Chemika, Buchs, Switzerland; 90 mM; pH 6.0) in D_2_O (Cambridge Isotope Laboratories, Andover, MA, USA)—containing 0.1% *w*/*w* trimethylsilylpropionic-2,2,3,3-d_4_ acid sodium salt (TMSP, Sigma-Aldrich, St. Louis, MO, USA)—and CD_3_OD (Sigma-Aldrich, St. Louis, MO, USA) (1:1). The mixture was vortexed at room temperature for 1 min, ultrasonicated (Elma Transsonic Digital, Hohentwiel, Germany) for 40 min, and then centrifuged (Beckman Allegra™ 64R, F2402H rotor; Beckman Coulter, Fullerton, CA, USA) at 13,000 rpm for 10 min. A volume of 0.60 mL was transferred to a 5-mm NMR tube and analyzed by NMR and LC-MS.

Partial purification of extracts was performed by dissolving the different dried extracts in water and partitioning them in ethyl acetate.

#### 2.3.1. NMR Experiments

NMR spectra were recorded at 25 °C on a 300.03 MHz for ^1^H and 75.45 MHz for ^13^C on a Bruker Fourier transform NMR. CD_3_OD was used as the internal lock. The ^1^H-NMR was acquired using a 1d-NOESY sequence to suppress the residual H_2_O signal. Each ^1^H-NMR spectrum consisted of 32 scans with the following parameters: acquisition time (AQ) = 4.54 s; relaxation delay (RD) = 2 s. Free induction decays (FIDs) were Fourier-transformed, and the resulting spectra were manually phased, baseline-corrected, and calibrated to TMSP. COSY, HSQC, and HMBC spectra were acquired using standard sequences from the Bruker library. On the partially purified fraction, NMR analyses were performed using standard zg30, HSQC, and HMBC sequences from the Bruker library.

#### 2.3.2. UHPLC-ESI-QqTOF HR MS/MS Experiments

Extracts were analyzed using a NEXERA UHPLC system (Shimadzu, Tokyo, Japan) and the Luna^®^ Omega C-18 column (Shimadzu, Tokyo, Japan). The linear gradient for separation purposes consisted of water (A) and acetonitrile (B), both with 0.1% formic acid. B eluent was held at 5% for 0.5 min and was allowed to reach 17.5% at 5 min; then, it was increased to 45% in 5 min and held at this % for the other 2 min. Finally, it was augmented to 95% in 1 min, and following another min at 95%, initial conditions were restored in 1 min, allowing for the system to re-equilibrate for 1 min. The flow rate was 0.5 mL/min. The injection volume was 2.0 μL. MS analysis was performed using the AB SCIEX Triple TOF^®^ 4600 (AB Sciex, Concord, ON, Canada), equipped with a DuoSpray^TM^ ion source, operating in negative ESI mode. A full scan TOF survey (170–1700 Da) and eight IDA (information-dependent acquisition) experiments were performed using the following parameters: curtain gas 35 psi; nebulizer gas 60 psi; heated gas 60 psi; ion spray voltage −4.5 kV; interface heater temperature 600 °C; declustering potential −80V; collision energy −45 ± 10 V. The instrument was controlled by Analyst^®^ TF 1.7.1 software. Data processing was carried out by PeakView^®^ software version 2.2.

### 2.4. Cell Lines and Culture Conditions

The cell lines HaCat, U-937, HL-60, and MCF-7 were obtained from the American Type Culture Collection (ATCC, Rockville, MD, USA). U-937 and HL-60 cells were grown in Roswell Park Memorial Institute (RPMI)-1640 medium (Gibco, New York, NY, USA) (Euroclone, Pero, Italy), while HaCat and MCF-7 in Dulbecco’s Modified Eagle Medium (DMEM) were grown at 37 °C in 5% CO_2_ atmosphere, then supplemented with 10% heat-inactivated fetal bovine serum (FBS), 1% l-glutamine, 1% ampicillin or streptomycin, and 0.1% gentamicin. All cell lines were treated with extract of **CWND**, **CBND**, and **WND** at different concentrations ranging from 1.25 mg/mL to 5 mg/mL from 24 h to 72 h. For the biological assays, *N. damascena* extracts were resuspended in dimethyl sulfoxide (DMSO) and used at the final concentrations indicated in each experiment. The DMSO was used as a negative control.

#### 2.4.1. MTT Assay

Cell vitality was determined by MTT assay. A total of 2 × 10^4^ cells/well were plated in a 96-well plate and treated, in triplicates, with extract of **WND**, **CWND**, and **CBND** at different concentrations and times. Thiazolyl Blue Tetrazolium Bromide [3-(4,5-dimethylthiazol-2-yl)-2,5-diphenyltetrazolium bromide] (MTT; Sigma-Aldrich) solution was added at 0.5 mg/mL. After 4 h, for the adhesion cells HaCat and MCF-7, the supernatant was simply removed, whereas, for the suspension cells U-937 and HL-60, the plates were first centrifuged. The purple formazan crystals were dissolved in isopropanol (Carlo Erba Reagents, Cornaredo, Italy), and the absorbance was read at a wavelength of 570 nm with a TECAN M-200 reader (Tecan, Männedorf, Switzerland) [25].

#### 2.4.2. FACS Analysis

The BD FACS Celesta flow cytometer from BD Biosciences was used to evaluate the percentage of cell death and the distribution of cells in the different phases of the cell cycle. After treatment with extracts, cells were harvested, and after washing with phosphate-buffered saline (1× PBS), cells were treated with PI buffer (1× PBS and 2 mg/mL propidium iodide) to evaluate the cell death rate. Subsequently, after a further wash with 1× PBS, the cells were treated with Cycle Buffer (1× PBS, 10% NP-40, 10% sodium citrate, and 2 mg/mL propidium iodide) for 15 min at room temperature to evaluate the percentage of cells in different phases of the cell cycle.

#### 2.4.3. Polymorphonuclear Leukocytes (PMN) Isolation

Whole blood was obtained with informed consent from healthy volunteers at the University “Federico II” in Naples, Italy. Polymorphonuclear leukocytes were isolated following the protocol described by Badalamenti et al. [26]. The isolated PMNs were measured in the presence or absence of extract of **WND**, **CWND**, and **CBND**, without or with Opsonised zymosan (OZ).

### 2.5. Reactive Oxygen Species (ROS) Generation and Antioxidant Enzymes Measured in PMN Cells

Dichlorofluorescein (DCF) assay was performed to quantify ROS generation, according to Manna et al. [27]. ROS quantity was monitored by fluorescence (excitation wavelength of 350 nm and an emission wavelength of 600 nm) on a microplate reader. Results were expressed as fluorescence intensity. A commercial kit (BioAssay System, San Diego, CA, USA) was used to determine superoxide dismutase (SOD) and catalase (CAT) enzymatic activity in PMN cells according to the manufacturer’s recommendations. The activity of enzymes was expressed as U/L [28]. The PMN was treated with extracts of **WND**, **CWND**, and **CBND** at the concentration of 0.5 mg/mL without or with OZ (0.5 mg/mL).

### 2.6. Statistics

All data and statistics were analyzed by GraphPad PrismTM 7.0 software (Graph PadPrism^TM^ Software Inc., San Diego, CA, USA). Values are mean ± standard deviation (SD) of biological triplicates.

## 3. Results

### 3.1. Chemical Characterization of the Extracts

The ^1^H-NMR spectra of the wild and cultivated *N. damascena* extracts (Figure S1) were analyzed. In all of the samples, traces of phenolic compounds were detected, but their relative abundance compared to the other components of the extracts was low. However, many signals in the region between 6.0 and 8.5 ppm were detected and suggested the presence of flavonoid derivatives [29,30]. Two doublets due to the meta-coupled protons of the A-ring of flavonoids (J = 2.0 Hz) were detected at *δ*_H_ 6.33 and 6.54, respectively. These two signals, which showed COSY correlations with each other (Figure S2), also showed HSQC correlations with carbons at *δ*_C_ 99.0 and 94.2, respectively (Figure S3). Furthermore, ABX and AA’BB’ aromatic systems were recognizable and suggested the presence of quercetin and kaempferol derivatives. The main quercetin derivative detected in the extracts was characterized by a doublet (J = 8.1 Hz) at *δ*_H_ 7.02 (*δ*_C_ 115.4), a doublet of a doublet at *δ*_H_ 7.56 (J = 8.1, 2.0 Hz; *δ*_C_ 122.0), and a doublet (J = 2.0 Hz) at *δ*_H_ 7.67 (*δ*_C_ 116.9). On the other hand, the main kaempferol derivative was characterized by two doublets (J = 8.5 Hz) at *δ*_H_ 7.00 (partially overlapped with the signal of quercetin at 7.02) and 8.10, correlating with each other in the COSY experiment. Long-range NMR experiments confirmed the presence of these flavonoids, but further discrimination of the different derivatives was not possible; the samples were, therefore, further analyzed by UHPLC-ESI QqTOF-MS/MS.

Beyond sweetener compounds (Figure S4), such as hexosyl hexitol (e.g., maltitol, C_12_H_24_O_11_), identifiably based on its [M-H]^−^ ion at *m*/*z* 343.1247, and the TOF MS/MS fragment ions at *m*/*z* 181.07 and 179.06, and *N*-(deoxyfructosyl)leucine ([M-H]^−^ ion at *m*/*z* 292.1402; C_12_H_23_NO_7_), the great part of the identified compounds in **WND**, **CWND**, and **CBND** extracts belong to flavonol glycosides (Table 1). In fact, the relative content of these compounds was 59.8, 87.1, and 60.5% in **WND**, **CWND**, and **CBND** extracts, respectively. As already suggested by NMR analyses, the derivatives of quercetin and kaempferol were the most representative. Quercetin glycosides accounted for 81.4, 74.5, and 83.2% of the extracts.

Compound **1**, with the [M-H]^−^ ion at *m*/*z* 949.2459, underwent, in the TOF-MS/MS experiment, neutral loss of 648.2 (162.05 × 4) Da to provide the radical aglycone at *m*/*z* 300.0281, according to glycosylation occurrence at C-3 position (Figure S5a). Thus, the quercetin tetrahexoside was likely quercetin 3-*O*-gentiotetroside [31].

Compounds **2**, **7**, **8**, and **16** were quercetin triglycosides (Figure S5b), mainly differing in their glyconic moiety. The deprotonated molecular ion of compound **2** underwent the neutral loss of 146.06 Da (deoxyhexose—H_2_O) to achieve the ion at *m*/*z* 625.14 or a homolytic cleavage to provide the radical ion at *m*/*z* 446.09. Both ions were in accordance with a dihexosyl moiety at the quercetin C-3 position, while deoxyhexose was positioned at C-7 carbon. According to this hypothesis, quercetin 3-*O*-sophoroside-7-*O*-rhamnoside was previously found in *N. sativa* [32]. The neutral loss of 486.15 Da from the [M-H]^−^ ion of compound **7**, and the high abundance of the TOF-MS/MS quercetin radical ion were in accordance with quercetin 3-*O*-trihexoside occurrence, while compound **8** was likely quercetin 3-*O*-hexosylpentoside-7-*O*-deoxyhexoside. In fact, the loss of dehydrated deoxyhexose was detectable, together with the radical ion at *m*/*z* 446.09. Finally, compound **16** was likely a quercetin 3-*O*-dihexosylpentoside.

Compounds **10**, **15**, and **20** were quercetin 3-*O*-diglycosides, showing as sugar moiety, dihexose (−324.11 Da), hexosylpentose (−294.10 Da), and hexosyldeoxyhexose (−308.11 Da), respectively (Figure S6), while quercetin hexoside (**21**) and quercetin deoxyhexoside (**31**) were the identified monoglycosyl derivatives. Compound **31** showed as the base peak of the ion at *m*/*z* 301.0368, according to glycosylation at the C-7 position.

Interestingly, hydroxymethyl glutaryl derivatives of the above-described quercetin glycosides, as well as acylated derivatives, were also identified (Figure S7). Compounds **9**, **13**, **18**, and **24** were hydroxymethyl glutaryl derivatives of compounds **2**, **7**, **8,** and **16**, respectively. The neutral losses of 62.00 Da (CO_2_ + H_2_O), 102.03 Da, and 144.04 Da suggested the presence of the hydroxymethyl glutaryl residue. The TOF-MS/MS spectra of compounds **9** and **13** highlighted that these neutral losses occurred from their relative deprotonated molecular ions and from the [M-H-(deoxyhexose-H_2_O)]^−^ ion, suggesting that hydroxymethyl glutaryl was located on C-3 glyconic residue. Analogously, a similar fragmentation pattern was shared from compound **23**, which was putatively quercetin (hydroxymethylglutaryl)hexosylpentoside.

Hydroxycinnamoyl quercetin triglycosides were also detected (Figure S8), distinguishable based on the hydroxycinnamoyl moiety in caffeoyl- (**19** and **22**), feruloyl- (**25** and **28**), and *p*-coumaroyl- (**26** and **27**) derivatives.

Similarly, kaempferol glycosides identified differed in the length of the glyconic moiety, and their identity (Figures S9 and S10). Briefly, compounds **3** and **6** were constitutional isomers, sharing the neutral loss of 146.05 Da and the formation through the loss of a dihexosyl radical of the radical form of kaempferol deoxyhexose. Compounds **11** and **14** were likely the hydroxymethyl glutaryl derivatives of the previous ones, while compounds **12** and **30** were tentatively kaempferol 3-*O*-trihexoside and its feruloyl derivative, respectively. Two kaempferol dihexosides (**4** and **17**) and kaempferol deoxyhexoside (**32**) were also recognized. Among kaempferol glycosides, kaempferol-3-*O*-[*β*-d-glucopyranosyl-(1 → 2)-*β*-d-galactopyranosyl-(1 → 2)-*β*-d-glucopyranoside was previously reported as an abundant constituent of *N. sativa*, but it was only at trace levels in other *Nigella* species, so much so that it was hypothesized as a marker for identifying *N. sativa*-based products [33]. Furthermore, myricetin dihexosylpentoside (**5**) and isorhamnetin dihexoside (**29**) were putatively identified.

**Figure 3:**
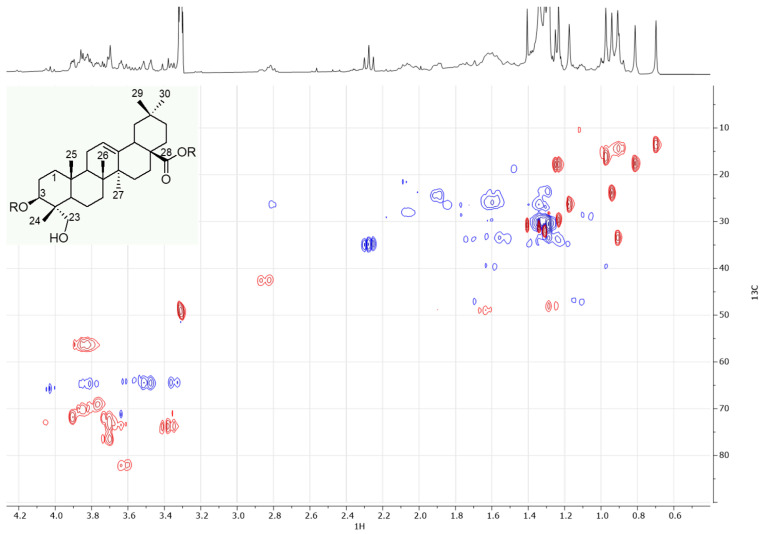
Detail of the HSQC spectrum of the ethyl acetate fraction obtained from the liquid/liquid partitioning of the crude polar extracts and structure of the hederagenin aglycone.

The other identified compounds were triterpene saponins, showing as aglycone hederagenin or oleanolic acid. This was confirmed both by the MS (Table 2, Figure S11) and NMR analyses. Concerning the NMR analyses, in order to simplify the identification of compounds in the mixture, a partial purification was carried out. While the more polar saponins were identified by MS (Table 2), the attention here was focused on the less polar and relatively abundant saponin, which was detected in the ethyl acetate fractions obtained from the partitioning of the whole extract with ethyl acetate/water. This fraction was analyzed by NMR. In the ^1^H and HSQC spectra (Figure 3 and Figure S12), a methyl singlet was detected at *δ*_H_ 0.70/*δ*_C_ 13.5. This signal showed long-range correlations (Figure S13) with two carbinol carbons at *δ*_C_ 81.8 and 64.0. The latter was identified as methylene, showing HSQC correlations with two diasterotopic protons at *δ*_H_ 3.36 and 3.50. These observations allowed us to attribute the signals at *δ*_H_ 0.70/*δ*_C_ 13.5 and that at *δ*_H_ 3.36–3.50/*δ*_C_ 64.0 to the positions 24 and 23 of the aglycone structure (Figure 3), respectively. The previously mentioned correlation observed for H-24 with the other carbinol carbon at *δ*_C_ 81.8 also suggested the presence of a hydroxyl group at the C-3 carbon of the aglycone. The H-24 protons also showed a long-range correlation with a quaternary carbon at *δ*_C_ 47.9, shared by another methyl signal at *δ*_H_ 0.97/*δ*_C_ 16.3. This allowed us to attribute the latter signals to the methyl-25. The signals belonging to the methyl-27 (*δ*_H_ 1.17/*δ*_C_ 26.3) were identified thanks to several correlations, with the diagnostic one being the correlation with the quaternary olefinic carbon (C-13) at *δ*_C_ 144.5. Methyl-26 was instead identified thanks to the correlations shared with methyl-25 and -27. Finally, the methyl-29 and methyl-30 at *δ*_H_ 0.90/*δ*_C_ 33.0 and at *δ*_H_ 0.94/*δ*_C_ 23.8, respectively, were identified based on their typically shared correlations. These data suggested that hederagenin was the aglycone of the most abundant saponin [34]. However, as highlighted in the MS analyses, the samples are characterized by the presence of several different saponins, and therefore, future studies could be aimed at the isolation and structural elucidation of these compounds.

### 3.2. Biological Properties

#### 3.2.1. Biological Effect of **CWND**, **CBND**, and **WND** in Hematological and Solid Cancer Cells

**Figure 4:**
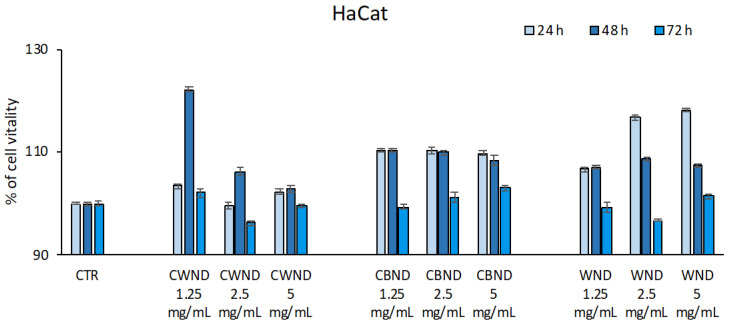
Cell vitality rates determined by MTT assay of HaCat cells after treatment with extracts of *N. damascena* with white flowers (**CWND**), blue flowers (**CBND**), and wild (**WND**) at the indicated concentrations and time. Values are mean ± standard deviation (SD) of biological triplicates.

To evaluate the biological activity of the extracts of three varieties of *N. damascena*, their antiproliferative effect was initially evaluated in normal keratinocytes (HaCaT). The HaCat cell line was treated with **CWND, CBND**, and **WND** extracts at three different concentrations (1.25, 2.5, and 5 mg/mL) for 24, 48, and 72 h. The results showed that the three *Nigella* extracts were not cytotoxic in normal cells because no significant effect on cell viability was displayed (Figure 4).

**Figure 5:**
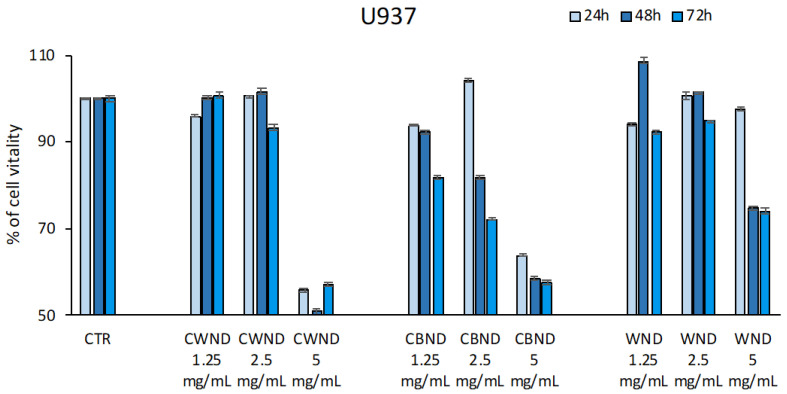

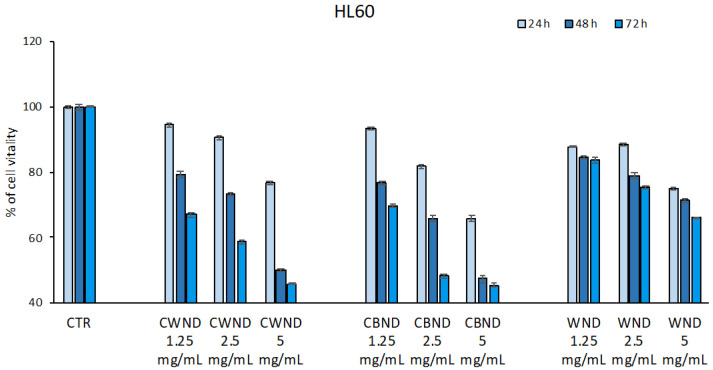
Cell vitality rates determined by MTT assay of U-937 and HL-60 cells after treatment with extracts of *N. damascena* with white flowers (**CWND**), blue flowers (**CBND**), and wild (**WND**) at the indicated concentrations and time. Values are mean ± standard deviation (SD) of biological triplicates.

On the contrary, in cancer cell lines, the three extracts showed a different biological efficacy. Specifically, in two leukemic cell lines, U-937 and HL-60, a reduction in cell viability was observed. In U-937, treatment with **CWND** was able to induce a reduction in cell viability (~40%) only at the highest concentration of 5 mg/mL; a similar effect was also observed after treatment with **CBND**, although a slight reduction was already appreciable at 1.25 and 2.5 mg/mL. A minor effect was achieved by **WND** treatment at 5 mg/mL. HL-60 cells appeared more sensitive to the treatments with **CWND** and **CBND**; a greater time-dose-dependent reduction in cell viability was observed, with a greater effect after 72 h at the maximum concentration used (~50%). No significant effect was obtained by **WND** treatment in this cell line (Figure 5).

**Figure 6:**
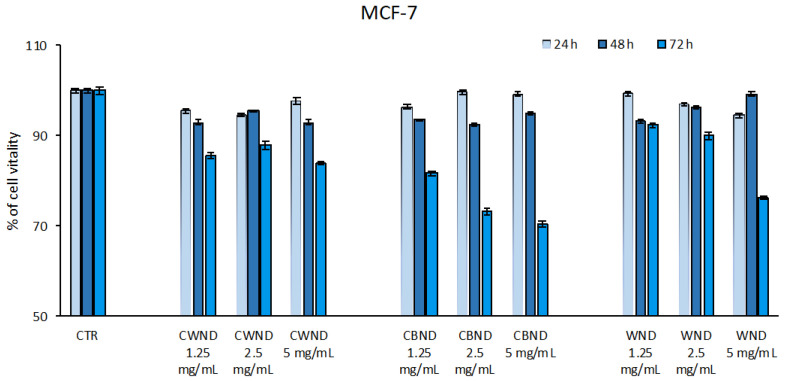
Cell vitality rates determined by MTT assay of MCF-7 cells after treatment with extracts of *N. damascena* with white flowers (**CWND**), blue flowers (**CBND**), and wild (**WND**) at the indicated concentrations and time. Values are mean ± standard deviation (SD) of biological triplicates.

In the MCF7 breast cancer cell line, the treatment with **CBND** induced a reduction in cell viability (~30%) only at 72 h for all three doses. A poor effect was noticed in both **CWND** and **WND** (Figure 6).

Taken together, the data suggest that **CWND** and **CBND** extracts have greater antiproliferative potential than **WND**. This effect is more evident in hematological tumor cell lines.

**Figure 7:**
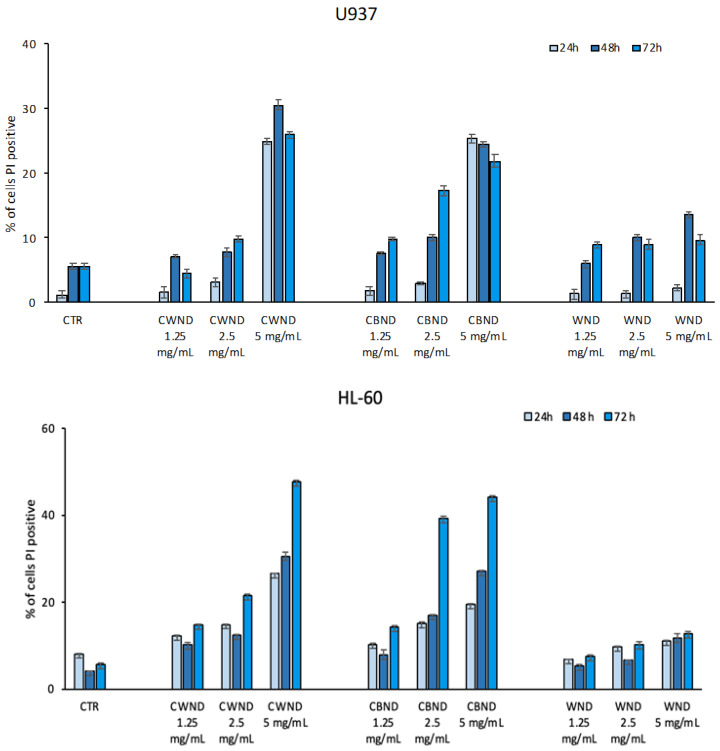
Cell death assay determined by FACS analysis of U-937 and HL-60 cells after treatment with extracts of *N. damascena* with white flowers (**CWND**), blue flowers (**CBND**), and wild (**WND**) at the indicated concentrations and time. Values are mean ± standard deviation (SD) of biological triplicates.

To better characterize the potential antiproliferative effect of the three extracts in the same cell lines, their biological effect on cell death and the cell cycle was tested. As shown in Figure 7, in line with what was observed from the cell viability analysis, we observed a greater effect on hematological cell lines than on epithelial cancer cell lines. The U-937 cells displayed an increase in the percentage of cell death (~30%) after treatment with **CWND** and **CBND** at a concentration of 5 mg/mL already after 24 h. The HL-60 cell line confirmed a greater sensitivity than U-937 to treatment with **CWND** and **CBND**, with a percentage of cell death up to ~50%. **WND** showed no significant effect (Figure 7).

**Figure 8:**
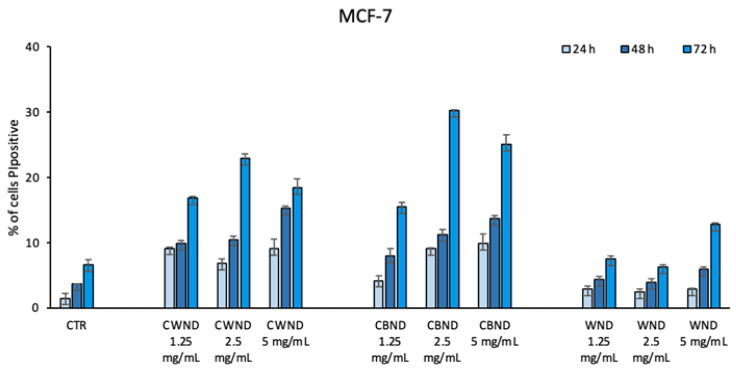
Cell death assay determined by FACS analysis of MCF-7 cells after treatment with extracts of *N. damascena* with white flowers (**CWND**), blue flowers (**CBND**), and wild (**WND**) at the indicated concentrations and time. Values are mean ± standard deviation (SD) of biological triplicates.

In MFC-7 cells, treatment with **CBND** at 2.5 and 5 mg/mL after 72 h induces an increase in cell death (~30%) (Figure 8).

The data obtained are in line with what was highlighted by the cell proliferation analysis conducted using the MTT assay.

**Figure 9:**
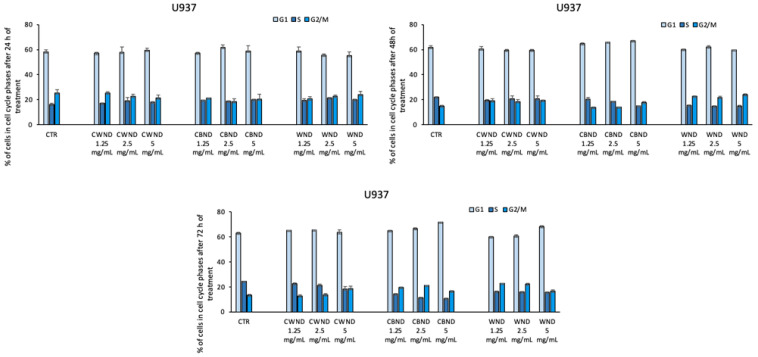
Cell cycle assay determined by FACS analysis of U-937 cells after treatment with extracts of *N. damascena* with white flowers (**CWND**), blue flowers (**CBND**), and wild (**WND**) at the indicated concentrations and time. Values are mean ± standard deviation (SD) of biological triplicates.

**Figure 10:**
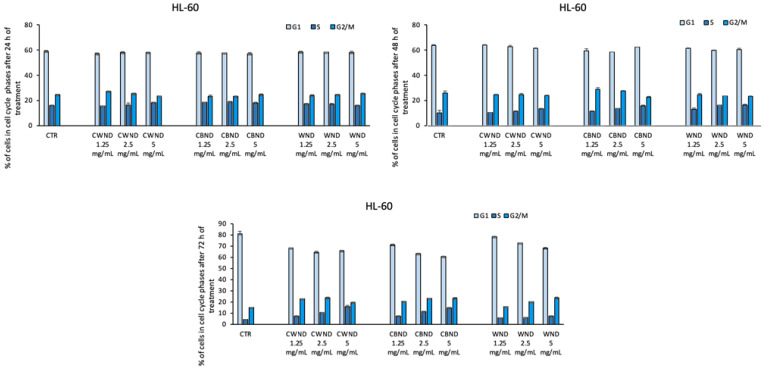
Cell cycle assay determined by FACS analysis of HL-60 cells after treatment with extracts *N. damascena* with white flowers (**CWND**), blue flowers (**CBND**), and wild (**WND**) at the indicated concentrations and time. Values are mean ± standard deviation (SD) of biological triplicates.

**Figure 11:**
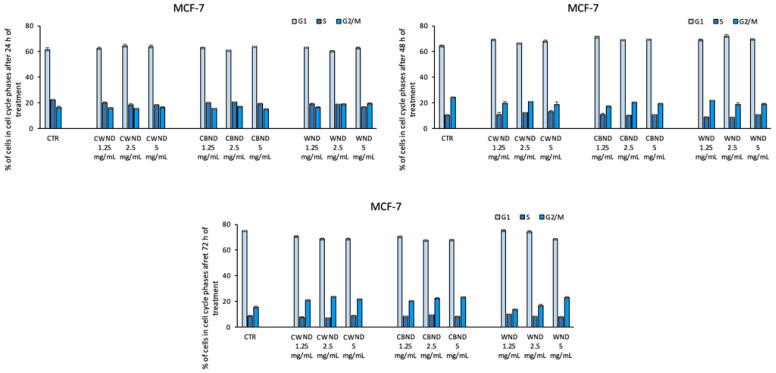
Cell cycle assay determined by FACS analysis of MCF-7 cells after treatment with extracts *N. damascena* with white flowers (**CWND**), blue flowers (**CBND**), and wild (**WND**) at the indicated concentrations and time. Values are mean ± standard deviation (SD) of biological triplicates.

Finally, cell cycle analysis also displayed that the treatment with **CWND, CBND**, and **WND** was not able to induce significant variations in cell cycle progression (Figure 9, Figure 10 and Figure 11).

#### 3.2.2. ROS Generation and Antioxidant Enzymes

**Figure 12:**
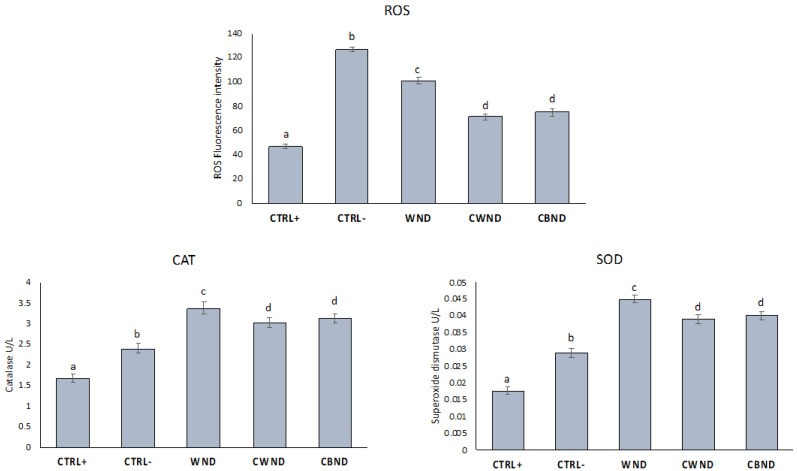
Reactive Oxygen Species (ROS) and the activities of antioxidant enzymes (superoxide dismutase; catalase) in PMN (Ctrl+), PMN with OZ (Ctrl−), PMN treated with white flowers (**CWND**), blue flowers (**CBND**), and wild (**WND**) at the concentration of 0.5 mg/mL and with OZ. Data were presented as mean and standard error and were analyzed with a paired *t*-test. Bars not accompanied by the same letter were significantly different at *p* < 0.05.

PMNs were treated with **CWND, CBND**, and **WND** extracts at a concentration of 0.5 mg/mL without or with OZ (0.5 mg/mL). As can be seen from Figure 12, ROS levels decreased after treatment with the three extracts compared to control. Furthermore, **CWND** and **CBND** extracts were more efficient than **WND** ones, while no significant differences were observed between the two cultivated plants. Furthermore, the extracts increased the activity of antioxidant enzymes compared to Ctrl+ and Ctrl−, with greater efficiency from the **CWND** and **CBND** extracts compared to **WND**.

In this study, we tried to elucidate how *N. damascena* extracts influenced two key aspects of cellular oxidative stress response: the generation of reactive oxygen species (ROS) and the activities of antioxidant enzymes. Specifically, they focused on polymorphonuclear leukocytes (PMN), a type of white blood cell crucial for immune defense and inflammation regulation.

Oxidative stress, characterized by an imbalance between ROS production and the body’s antioxidant defenses, plays a pivotal role in various pathological conditions, including inflammation and cancer. Antioxidant enzymes such as superoxide dismutase and catalase act as frontline defenders against ROS-mediated damage by neutralizing harmful free radicals.

## 4. Discussion

Spectroscopic studies carried out with the main aid of two-dimensional NMR spectra, coupled with the mass spectrometry technique, confirmed the clear presence of flavonoids and triterpene saponins (Table 1 and Table 2). Regarding triterpenoids, four have been described for the first time from the methanolic extract of the aerial parts of several *N. damascena* plants cultivated in Japan. These triterpene glycosides, also called nigellosides A-D, were identified with eight other triterpene glycosides known in the literature [34]. The latter isolated saponins are not chemotaxonomic markers of the *Nigella* genus because they have been isolated from various species such as *Blighia welwitschii* (Hiern) Radlk. [35], *Dipsacus azureus* Schrenk [36], *Schefflera rotundifolia* [37], *Patrinia scabiosaefolia* Link, a Chinese drug whose extract has shown hepatotoxic activities [38], as well as anti-tumor and anti-inflammatory effects [39], *Lonicera japonica* Thunb. [40], and *Anemone anhuiensis* roots [41]. While there are few scientific studies in which the isolation of triterpenes from *N. damascena* has been reported [34], the situation is different for the relative *N. sativa*. Recently, *N. sativa* aerial parts were phytochemically investigated to isolate, among the others, a new compound, namely, 3*β*,23,28-trihydroxyolean-12-ene-3-*O*-*α*-l-arabinopyranosyl-(1 → 4)-*α*-l rhamno pyranosyl-(1 → 4)-*β*-d-gluco-pyranoside [42]. In this work, spectroscopic data allowed for the provisional and qualitative identification of eleven compounds (**1**′–**11**′), which mainly shared glycosylation at the C-3 carbon and esterification at the C-28 carbon of the aglycone core. This is totally in line with the compounds identified by Yoshimitsu et al. [34], who reported the glycosylation site on C-3 and a C_12_-C_13_ double bond. Their relative quantitation highlighted that these compounds were poorly abundant in **CWND**, while they were similarly present in **WND** and **CBND** extracts. The TOF-MS/MS spectra of all the compounds are reported in Figure S11.

The blue, pink, and light-blue color of *N. damascena* flowers is due to the presence of various flavonoids, in particular, glycosylated anthocyanins. The flavonoids contained in the blue flowers of *N. damascena*, grown in Japan, were studied and led to the isolation of the anthocyanin petunidin 3-*O*-(6′-*O*-*α*-rhamnopyranosyl-2′-*O*-*β*-xylopyranosyl-*β*-glucopyranosides [43]. From the HPLC-MS analysis of the flowers of “Miss Jekyll Rose Shade”, structure, such as the cyanidin 3-*O*-[2-*O*-(*β*-glucopyranosyl)-6-*O*-(*α*-rhamnopyranosyl)-*β*-glucopyranoside]) [44], was identified, while from the flowers of two cultivars, “Miss Jekyll Blue” and “Miss Jekyll White”, the presences of delphinidin 3-[2-(xylosyl)-6-(rhamnosyl)-glucoside] and 7-*O*-methyldelphinidin were highlighted [44]. It is, therefore, essential to carry out further studies to confirm the exact molecular structure of the compounds identified here and understand possible differences between the cultivars already studied.

Different studies in the literature have investigated the anti-tumor potential of *N. damascena* essential oil and extract. This is because it would seem that the activity is mainly due to the presence of the sesquiterpene hydrocarbon *β*-elemene, whose presence can reach up to 73.0% [45,46]. It seems capable of modifying the permeability of the cell membrane, increasing cellular absorption of the drug. This compound can hardly be present in these polar extracts, so the demonstrated activity is to be found toward the identified triterpene structures or in the presence of dolabellane metabolites. For example, 3-*O*-*β*-*D*-glucopyranosyl-(1→3)-*α*-*L*-rhamnopyranosyl-(1→2)-*α*-*L*-arabinopyranosyl hederagenin 28-*β*-*D*-glucopyranosyl ester, a glycosidic triterpene isolated from the methanolic extract of *N. damascena* aerial parts, presented excellent antiproliferative activity against three different murine and human cell lines such as J774. A1, WEHI-164 e HEK-293 (IC_50_ = 0.51–1.8 μM) [37], while dipsacoside B did not show relevant activity neither against A549 and SGC-7901 tumor cells nor against the human liver cell line (HL-7702) [47]. In turn, dolabellane, a compound belonging to the sesquiterpene’s class, isolated from the aerial parts of *N. glandulifera* seeds but also presented in the aerial parts, showed moderate cytotoxic activity, reducing the viability of T98G, U87, U251, and GL261 glioma cancer cell lines by only 29% [48].

It has been widely demonstrated that the biological effects of extracts are the result of both the different compositions of the extracts themselves and the synergistic action of the compounds contained in them. It is also known that the composition and concentration of the different bioactive compounds in plants and, consequently, the biological effectiveness of the extracts can be influenced by numerous factors, among which, certainly, the genotype and environmental factors play an important role [49]. The data obtained in this study revealed that the three tested extracts had different antiproliferative potentials. In particular, **CWNB** and **CBND** showed a stronger anti-tumor effect than **WND**, especially against U937 and HL-90 tumor cell lines, and this effect could be ascribed to the peculiar content of bioactive compounds present in each extract. However, in our case, the observed differences in the bioactivity of the extracts cannot be attributed to a single metabolite or class of compounds. The synergistic effect of the different compounds on the antiproliferative activity is very likely, and further efforts and energy must be made to confirm the real mechanism of action by isolating the different metabolites.

A study by Salmani et al. [50] examined the cytotoxic effects of different *Nigella damascena* seed extracts on breast cancer cells (MDA-MB-231 and MCF-7) and colon cancer cells (HT-29). They found that the ethyl acetate extract exhibited significant cytotoxic activity against both breast and colon cancer cell lines, suggesting its potential as a therapeutic agent against these cancers [50]. Another study conducted by Karimi et al. [51] investigated the antiproliferative effects of *Nigella damascena* seed extract on human glioblastoma multiforme (GBM) cell lines. The results demonstrated that the extract inhibited the proliferation of GBM cells and induced apoptosis, indicating its potential as a therapeutic agent for the treatment of GBM [51].

By investigating the effects of *N. damascena* extracts on ROS production and the activities of these antioxidant enzymes in PMN, this study aimed to shed light on the potential therapeutic properties of the extract in mitigating oxidative stress-related disorders. Understanding how natural compounds like *N. damascena* modulate oxidative stress pathways in immune cells could pave the way for developing novel treatments targeting inflammatory and oxidative stress-related conditions.

The antioxidant activity of natural extracts is very often linked to the presence of hydrolyzed aromatic compounds [52] and to the presence of phenolic acids [53]. Alu’datt and colleagues [54] demonstrated that several protein fractions obtained from *N. damascena* aerial parts, characterized by a notable presence of linked phenolic compounds, were able to increase the content of free phenolics, demonstrating excellent antioxidant activity. The phenolic content, influenced by a possible qualitative diversity of compounds such as polyphenols and flavonoids present in **CWND**, **CBND**, and **WND**, influences the antioxidant activity of the investigated extracts. Further studies are needed to evaluate quantitative differences in the studied extracts, isolating the individual metabolisms present.

Antioxidants play a crucial role in protecting cells from oxidative stress-induced damage, which is implicated in various diseases, including cancer and neurodegenerative disorders. Several studies have explored the antioxidant activity of *N. damascena* extracts. A study conducted by Hosseinzadeh et al. [55] evaluated the antioxidant activity of *N. damascena* seed extract using various in vitro assays, including DPPH (2,2-diphenyl-1-picrylhydrazyl) radical scavenging and ferric reducing antioxidant power (FRAP) assays. The results demonstrated significant antioxidant activity of the seed extract, suggesting its potential in scavenging free radicals and reducing oxidative stress [55].

The use of natural agents to regulate antioxidant properties and tumorigenesis is increasing. For example, the anti-tumor potential of other plants of the *Nigella* genus has also been highly described. In fact, the anti-tumor effects of *Nigella sativa* have been characterized, together with antiproliferative, pro-apoptotic, antioxidant, cytotoxic, anti-mutagenic, and antimetastatic properties [56].

The main molecular mechanisms of action implicated in the described activities are known, and the results reported over the last two decades strongly suggest that *N. sativa* fractions could serve, alone or in combination with known chemotherapeutic drugs, as effective agents to control the oxidative procession and the onset of the tumor, growth, and metastasis of a wide range of tumors [57].

Our preliminary data, and the evidence in the literature of the antiproliferative and antioxidant activities of *Nigella* extracts, suggest that further studies are certainly necessary to shed more light on the molecular and cellular aspects relating to the mechanisms underlying the *N. damascena* properties. We hope that further research efforts will clarify the mechanisms involved in its potential suppressive role in tumorigenesis and cancer. Experimental evidence suggests potent anticancer effects of *N. damascena* extracts, but preventive and clinical studies directly indicating the anticancer potential of its extracts are still lacking.

## 5. Conclusions

In this study, the chemical profile of *Nigella damascena* plants, subjected to different pedoclimatic conditions, was evaluated. Based studies performed via 1D- and 2D-NMR and UHPLC-ESI-QqTOF HR MS/MS have demonstrated the presence of metabolites such as sugars, free amino acids, organic acids, saponins, and flavonoid compounds. A qualitative difference was found between the three extracts studied regarding glycosylated triterpenes. Their relative quantitation highlighted that these compounds were poorly abundant in **CWND**, while they were similarly present in **WND** and **CBND** extracts.

The biological results suggest that the three *Nigella* extracts are able to induce antiproliferative effects in cancer cell lines, with a major effect in hematological malignant cells and any effects in normal cells. In the antiproliferative tests, **CWNB** and **CBND** displayed a stronger anticancer effect than **WND**. This effect was more evident in hematological cancer cell lines U-937 and HL-60.

At present, there is ambiguity regarding whether certain beneficial effects of numerous natural compounds, like their anticancer properties, require direct transcriptional activity or are primarily influenced by epigenetic mechanisms. Consequently, additional research is warranted to assess whether these observed biological activities might stem from potential epigenetic effects exerted by secondary metabolites, as evidenced in prior studies on other natural compounds [58]. This preliminary study is the basis for further research in which we will try to isolate the preponderant metabolites individually, thus evaluating their contribution to the anti-tumor and antioxidant activity.

## Figures and Tables

**Table 1:** Chromatographic and TOF MS data of flavonoid glycosides tentatively identified in **WND**, **CBND**, and **CWND** *N. damascena* extracts. RDB = Ring Double Bonds.

Peak	Rt	Tentative Assignment	Molecular Formula	[M-H]^−^ *Found*	[M-2H]^2−^	RDB	Error (ppm)
**1**	3.791	Quercetin tetrahexoside	C_39_H_50_O_27_	949.2459		15	−0.8
**2**	4.010	Quercetin dihexoside deoxyhexoside	C_33_H_40_O_21_	771.1993		14	0.5
**3**	4.269	Kaempferol dihexoside deoxyhexoside (1)	C_33_H_40_O_20_	755.2046		14	0.8
**4**	4.308	Kaempferol dihexoside	C_27_H_30_O_16_	609.1460		13	−0.2
**5**	4.315	Myricetin dihexosylpentoside	C_32_H_38_O_22_	773.1751		14	−4.0
**6**	4.373	Kaempferol dihexoside deoxyhexoside (2)	C_33_H_40_O_20_	755.2046		14	−1.6
**7**	4.409	Quercetin trihexoside	C_33_H_40_O_22_	787.1940		14	0.2
**8**	4.450	Quercetin hexosylpentoside deoxyhexoside	C_32_H_38_O_20_	741.1884		14	0.9
**9**	4.452	Quercetin (hydromethylglutaryl) dihexoside deoxyhexoside	C_39_H_48_O_25_	915.2399		16	−1.4
**10**	4.562	Quercetin dihexoside	C_27_H_30_O_17_	625.1426		13	2.5
**11**	4.740	Kaempferol (hydromethylglutaryl) dihexoside deoxyhexoside (1)	C_39_H_48_O_24_	899.2499		16	1.6
**12**	4.883	Kaempferol trihexoside	C_33_H_40_O_21_	771.1979		14	−1.3
**13**	4.885	Quercetin (hydromethylglutaryl) hexosylpentoside deoxyhexoside	C_38_H_46_O_24_	885.2285		16	−2.4
**14**	4.889	Kaempferol (hydromethylglutaryl) dihexoside deoxyhexoside (2)	C_39_H_48_O_24_	899.2452		16	−1.2
**15**	4.954	Quercetin hexosylpentoside	C_26_H_28_O_16_	595.1308		13	0.6
**16**	4.957	Quercetin dihexosylpentoside	C_32_H_38_O_21_	757.1839		14	0.8
**17**	5.032	Kaempferol dihexoside	C_27_H_30_O_16_	609.1463		13	0.3
**18**	5.113	Quercetin (hydromethylglutaryl) trihexoside	C_39_H_48_O_26_	931.2346		16	−1.4
**19**	5.231	Quercetin caffeoyltrihexoside (1)	C_42_H_46_O_25_	949.2246	474.1091	24	−1.0
**20**	5.339	Quercetin hexosyldeoxyhexoside	C_27_H_30_O_16_	609.1463		13	0.3
**21**	5.471	Quercetin hexoside	C_21_H_20_O_12_	463.0881		12	−0.2
**22**	5.505	Quercetin caffeoyltrihexoside (2)	C_42_H_46_O_25_	949.2248	474.1094	24	−0.8
**23**	5.560	Quercetin (hydroxymethylglutaryl) hexosylpentoside	C_32_H_36_O_20_	739.1730		15	−1.9
**24**	5.572	Quercetin (hydroxymethylglutaryl) dihexosylpentoside	C_38_H_46_O_25_	901.2248		16	−0.8
**25**	5.861	Quercetin feruloyltrihexoside (1)	C_43_H_48_O_25_	963.2393		20	−2.0
**26**	6.065	Quercetin coumaroyltrihexoside (1)	C_42_H_46_O_24_	933.2306	466.1118	20	−1.9
**27**	6.058	Quercetin coumaroyltrihexoside (2)	C_42_H_46_O_24_	933.2303		20	−0.4
**28**	6.142	Quercetin feruloyl trihexoside (2)	C_43_H_48_O_25_	963.2393		20	−0.5
**29**	6.209	Isorhamnetin dihexoside	C_28_H_32_O_16_	623.1623		13	0.9
**30**	6.338	Kaempferol feruloyl trihexoside	C_43_H_48_O_24_	947.2463		20	0
**31**	6.941	Quercetin deoxyhexoside	C_21_H_20_O_11_	447.0942		12	2.0
**32**	7.525	Kaempferol deoxyhexoside	C_21_H_20_O_10_	431.0993		12	2.2

When two isomers of the same compound are detected and cannot be distinguished from each other, they are indicated by a number in brackets.

**Table 2:** Chromatographic and TOF MS data of triterpenoid saponins tentatively identified in **WND**, **CBND**, and **CWND** *N. damascena* extracts. RDB = Ring Double Bonds.

Peak	Rt	Tentative Assignment	Molecular Formula	[M-H]^−^ *Found*	[M-H]^2−^ *Found*	RDB	Error (ppm)
**1′**	7.734	3- hexosyl deoxyhexosyl hederagenin 28-hexosyl ester	C_48_H_78_O_18_	941.5114		10	−0.1
**2′**	8.061	3-hexosyl pentosyl hederagenin 28-hexosyl ester	C_47_H_76_O_18_	927.4963		10	0.4
**3′**	8.350	3-hexosyl deoxyhexosyl pentosyl hederagenin 28-dihexosyl ester	C_61_H_100_O_31_	1235.6059	663.3057	10	0.8
**4′**	8.490	3-deoxyhexosyl pentosyl hederagenin 28-hexosyl ester	C_47_H_76_O_17_	911.5018		10	0.9
**5′**	8.928	3-hexosyl deoxyhexosyl pentosyl oleanolic acid	C_47_H_76_O_16_	895.5038		10	−1.4
**6′**	9.140	3-deoxyhexosyl pentosyl oleanolic acid 28-hexosyl ester	C_47_H_76_O_16_	895.5055		10	−0.6
**7′**	9.416	3-deoxyhexosyl pentosyl oleanolic acid 28-dihexosyl ester	C_53_H_86_O_21_	1057.5585		11	−0.4
**8′**	9.813	3-hexosyl deoxyhexosyl hederagenin 28-hexosyl ester	C_48_H_78_O_18_	941.5119		10	0.4
**9′**	10.365	3-hexosyl deoxyhexosyl hederagenin pentosyl ester (1)	C_47_H_76_O_17_	911.5000		10	−1.1
**10′**	10.502	3-hexosyl deoxyhexosyl hederagenin pentosyl ester (2)	C_47_H_76_O_17_	911.5017		10	0.8
**11′**	10.769	3-hexosyl deoxyhexosyl dehydrohederagenin pentosyl ester	C_47_H_74_O_17_	909.4855		10	0.2

When two isomers of the same compound are detected and cannot be distinguished from each other, they are indicated by a number in brackets.

## Data Availability

All data and materials are available upon request from the corresponding author.

## Supplementary Materials

The following supporting information can be downloaded at https://www.mdpi.com/article/10.3390/antiox13040402/s1, Figure S1. ^1^H-NMR spectra, acquired at 300 MHz in CD_3_OD, of **WND**, **CBND**, and **CWND** *N. damascena* extracts; Figure S2. COSY spectrum of **WND** extract: aromatic region; Figure S3. HSQC spectrum of **WND** extract: aromatic region; Figure S4. TOF-MS/MS spectra of hexosyl hexitol (**a**) and *N*-(deoxyfructosyl)leucine (**b**), tentatively identified in *N. damascena* extracts. Theoretical *m*/*z* values are reported below each structure; Figure S5. TOF-MS/MS spectra of (a) quercetin tetrahexoside (**1**) and (b) quercetin triglycosides (i) **2**, (ii) **7**, (iii) **8**, and (iv) **16**. Theoretical *m*/*z* values are reported below each structure; Figure S6. TOF-MS/MS spectra of quercetin diglycosides (a–c) and monoglycosides (d,e); Figure S7. TOF-MS/MS spectra (i) of quercetin hydroxymethylglutaryl triglycosides **9** (a), **13** (b), **18** (c), and **24** (d). The fragmentation pattern hypothesized for compound **9** is depicted (ii); theoretical *m*/*z* values are below each structure; Figure S8. TOF-MS/MS spectra of quercetin hydroxycinnamoyl glycosides **19** (a), **22** (b), **25** (c), **28** (d), **26** (e), and **27** (f); Figure S9. TOF-MS/MS spectra of kaempferol triglycosides **3** (a), **6** (b), **12** (c); the hydroxymethylglutaryl derivatives **11** (d), and **14** (e), and kaempferol feruloyl trihexoside **30** (f); Figure S10. TOF-MS/MS spectra of kaempferol glycosides **4** (a), **17** (b), and **32** (c), and of myricetin triglycoside (**5**) (d) and isorhamnetin dihexoside (**29**) (e); Figure S11. TOF-MS/MS spectra of triterpene saponins **1′**–**11′** (a–l). The TIC (Total Ion Chromatogram) region in which the compounds eluted is shown in the upper part of the figure; Figure S12. HSQC spectrum of the ethyl acetate fraction obtained from the L/L partitioning of the crude polar extract; Figure S13. HMBC spectrum of the ethyl acetate fraction obtained from the L/L partitioning of the crude polar extract.
