# Supplementary material for: Phytochemical Characterization, Antioxidant, and Anti-Proliferative Activities of Wild and Cultivated Nigella damascena Species Collected in Sicily (Italy)
Source: Antioxidants (Basel). 2024 Mar 27;13(4):402. doi: 10.3390/antiox13040402 (PMC11047655; doi:10.3390/antiox13040402)
Supplement: Supplementary file 1 [file antioxidants-13-00402-s001.zip › antioxidants-2876541-supplementary.pdf]

Supplementary Materials

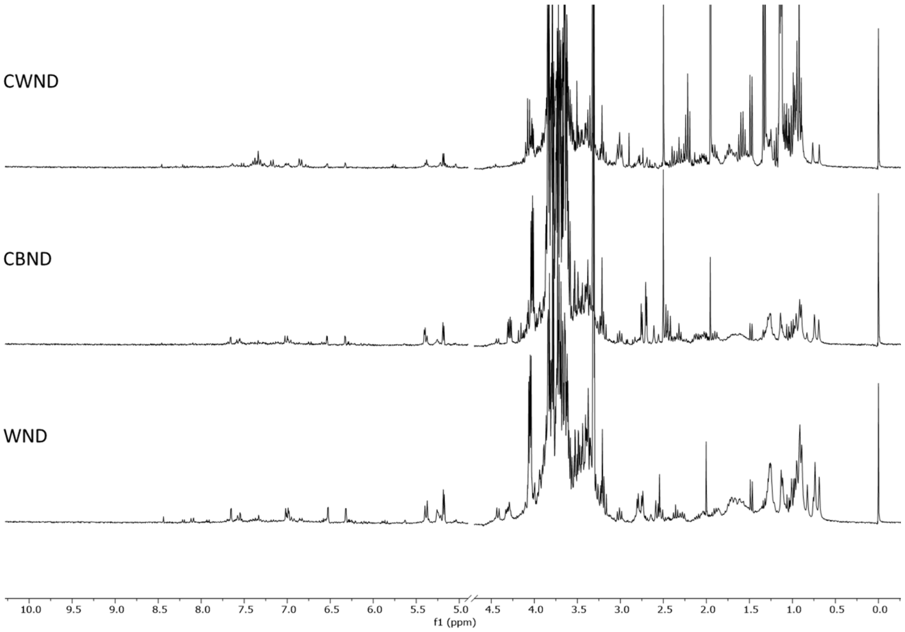

Figure S1. ^{1}H-NMR spectra, acquired at 300 MHz in CD_3OD, of **WND**, **CBND**, and **CWND** *N. damascena* extracts.

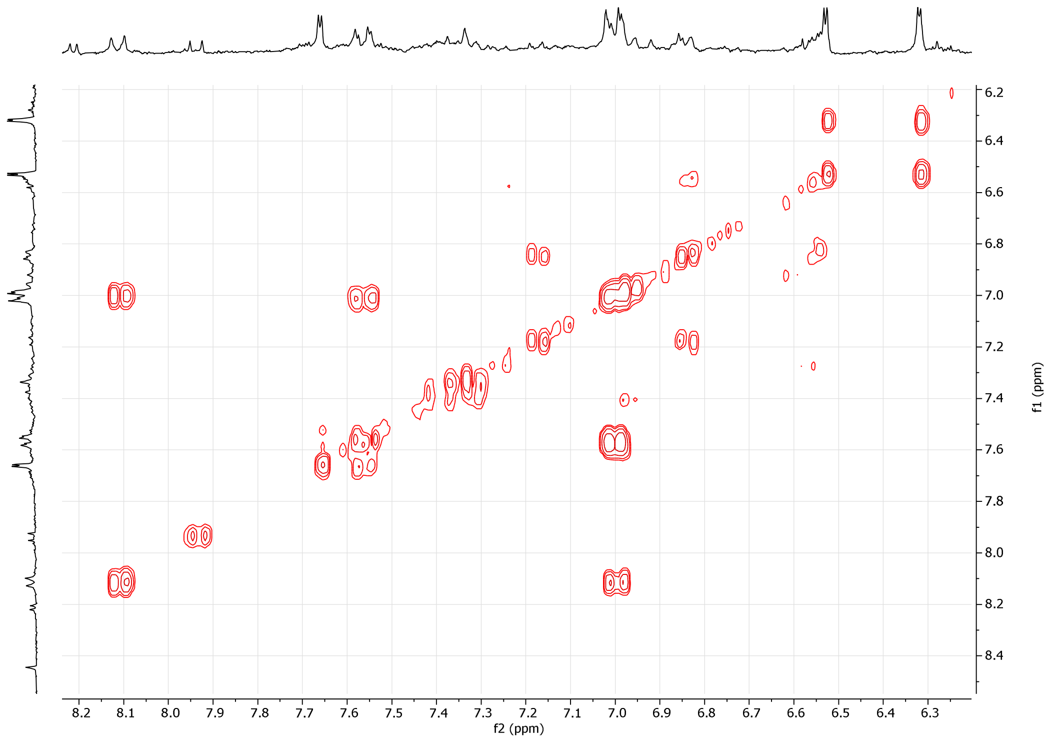

Figure S2. COSY spectrum of **WND** extract: aromatic region.

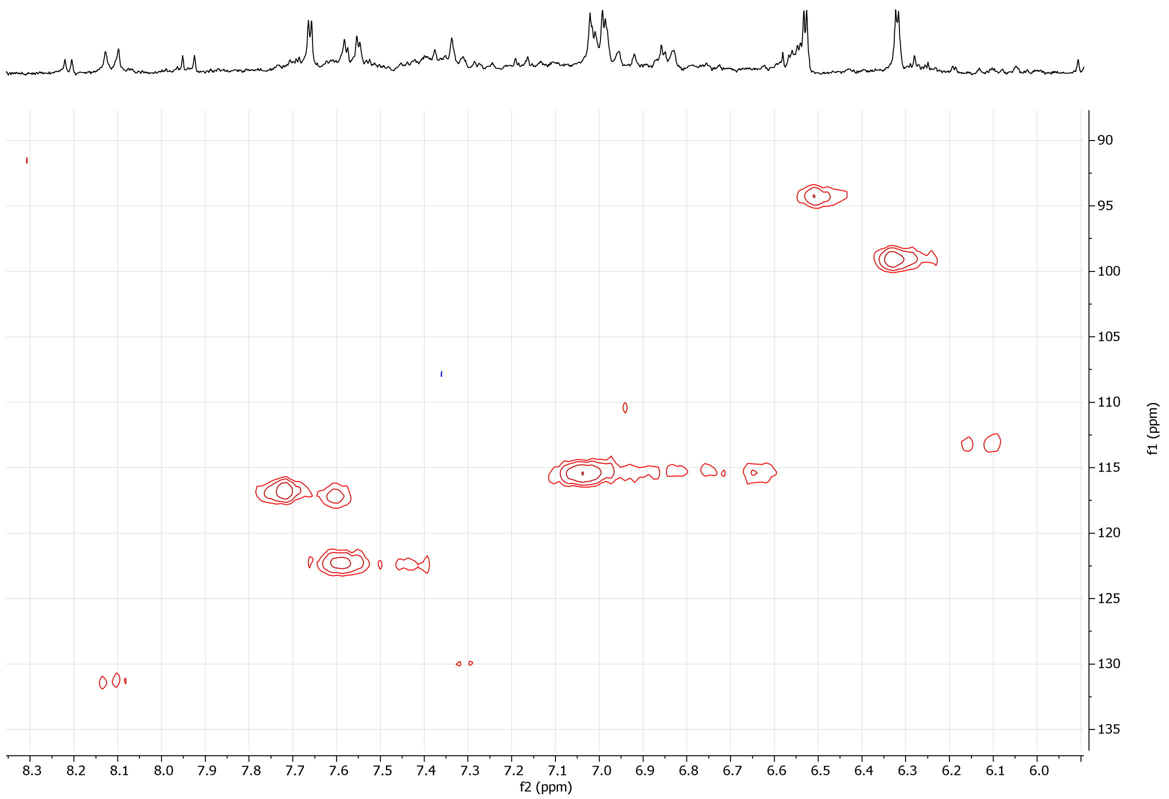

Figure S3. HSQC spectrum of **WND** extract: aromatic region.

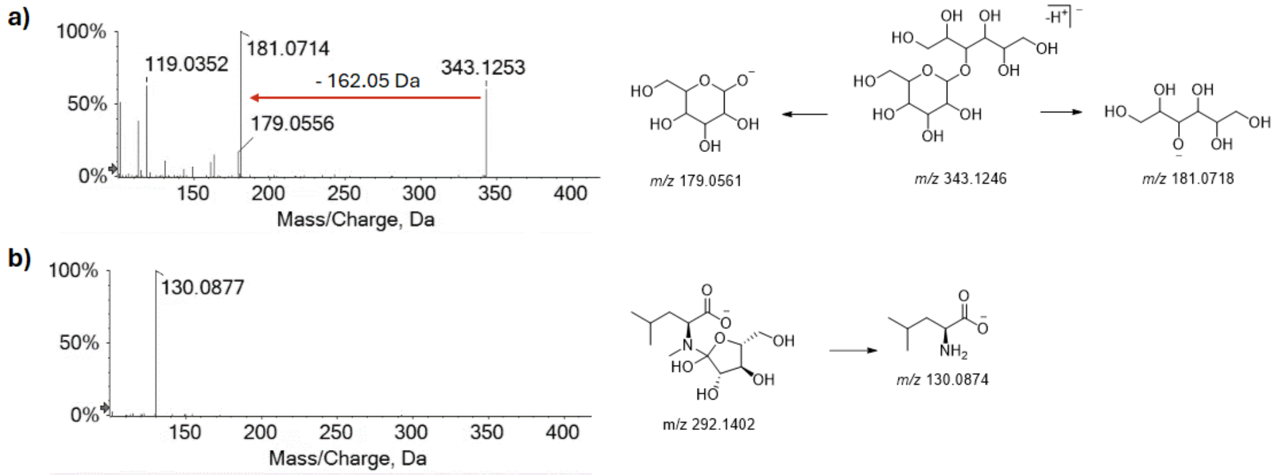

Figure S4. TOF-MS/MS spectra of hexosyl hexitol (a) and N-(deoxyfructosyl)leucine (b), tentatively identified in *N. damascena* extracts. Theoretical *m/z* values are reported below each structure.

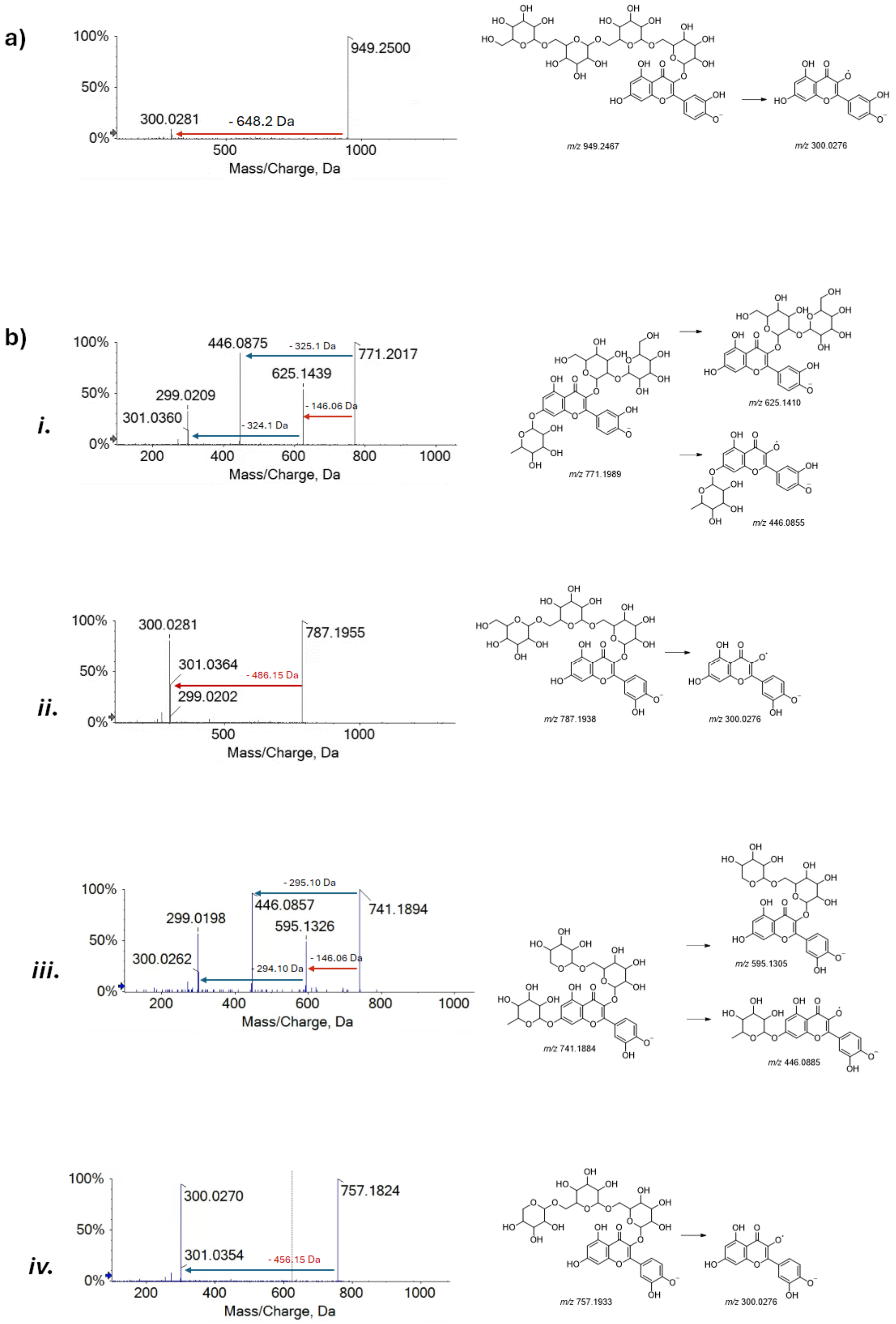

Figure S5. TOF-MS/MS spectra of (a) quercetin tetrahexoside (**1**), and (b) quercetin triglycosides (i). **2**; (ii). **7**; (iii). **8**, and (iv). **16**. Theoretical *m*/*z* values are reported below each structure.

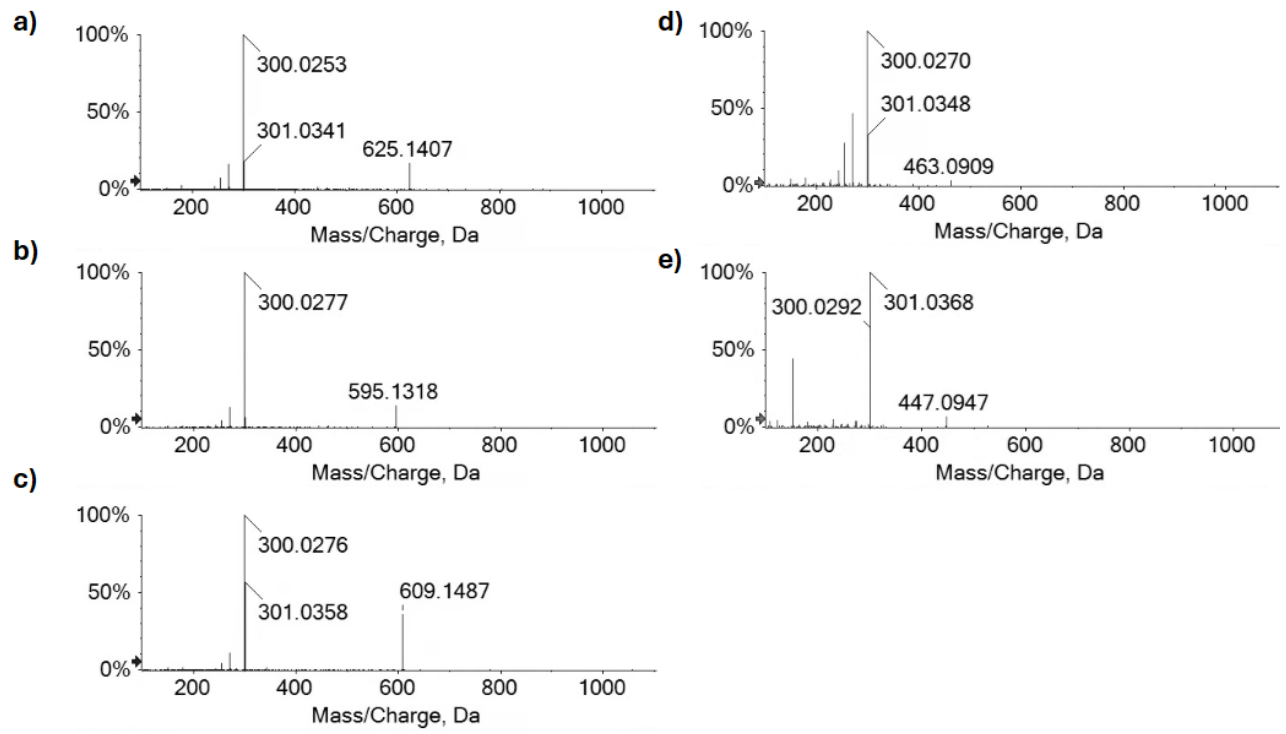

Figure S6. TOF-MS/MS spectra of quercetin diglycosides (a-c), and monoglycosides (d, e).

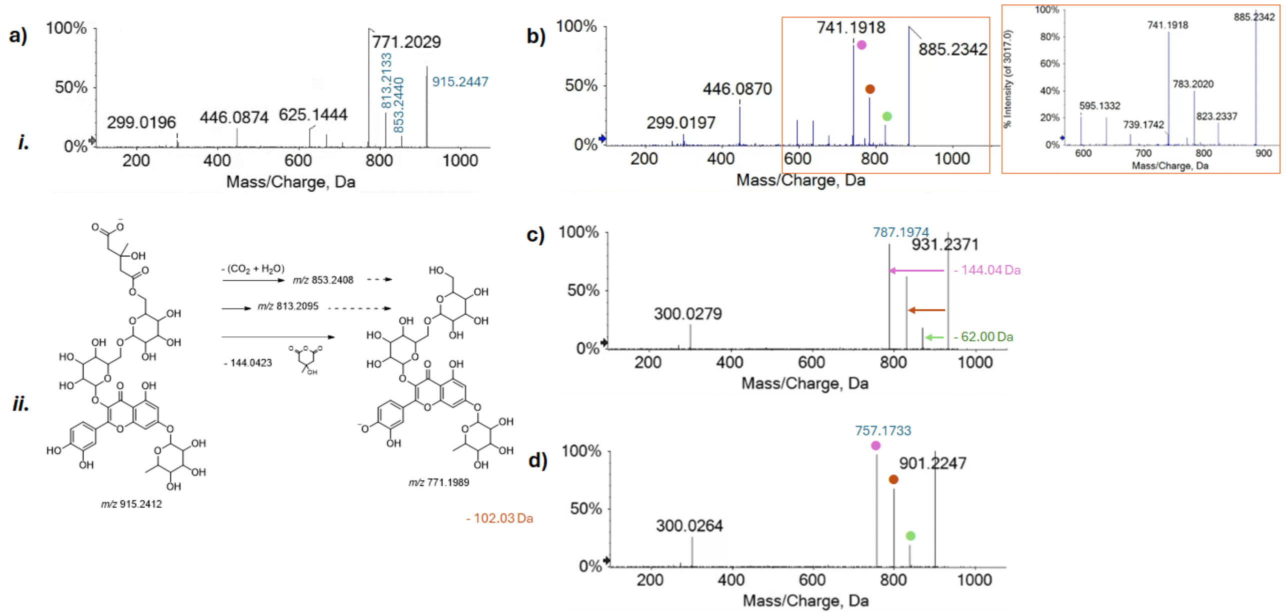

Figure S7. TOF-MS/MS spectra (i) of quercetin hydroxymethylglutaryl triglycosides **9** (a), **13** (b), **18** (c), and **24** (d). The fragmentation pattern hypothesized for compound **9** is depicted (ii); theoretical *m*/*z* values are below each structure.

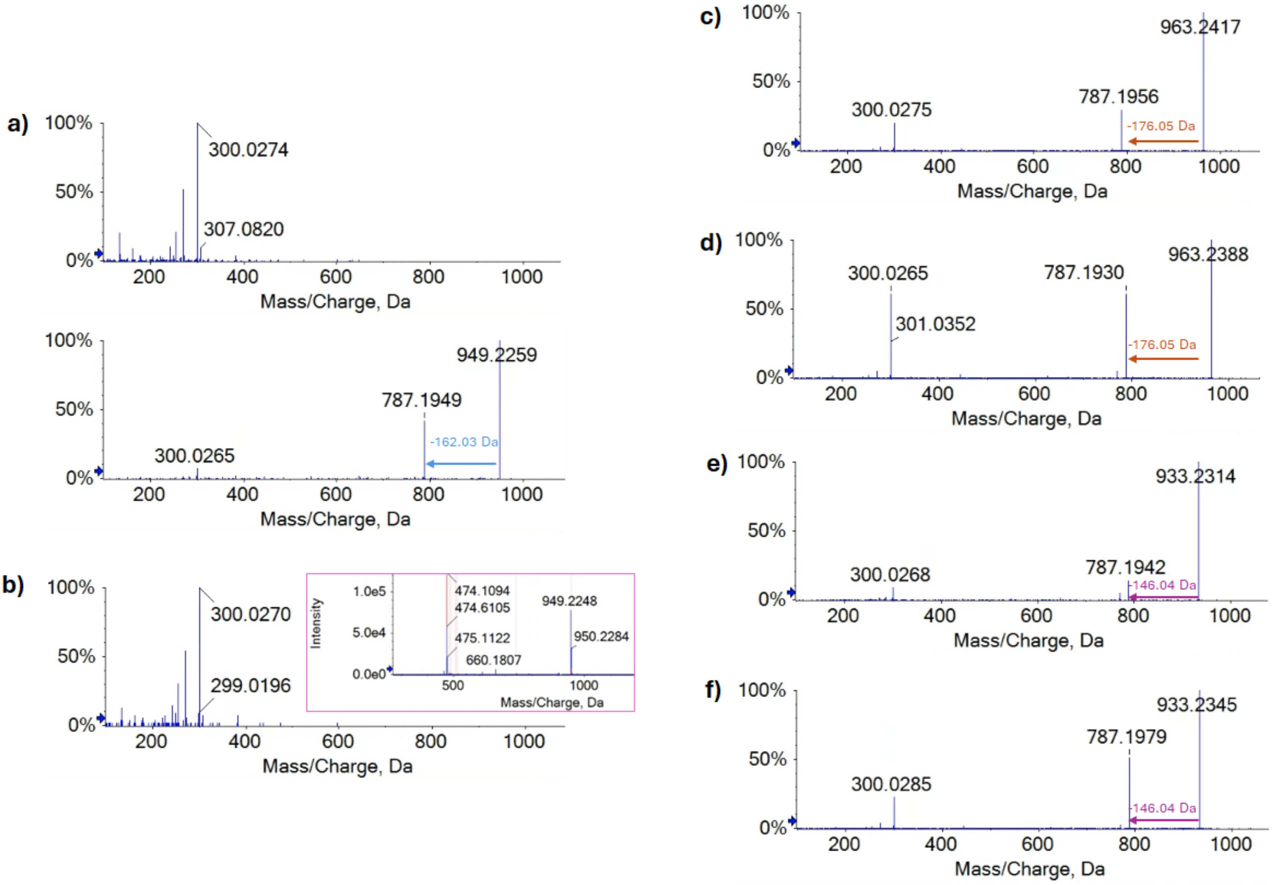

Figure S8. TOF-MS/MS spectra of quercetin hydroxycinnamoyl glycosides **19** (a), **22** (b), **25** (c), **28** (d), **26** (e), and **27** (f).

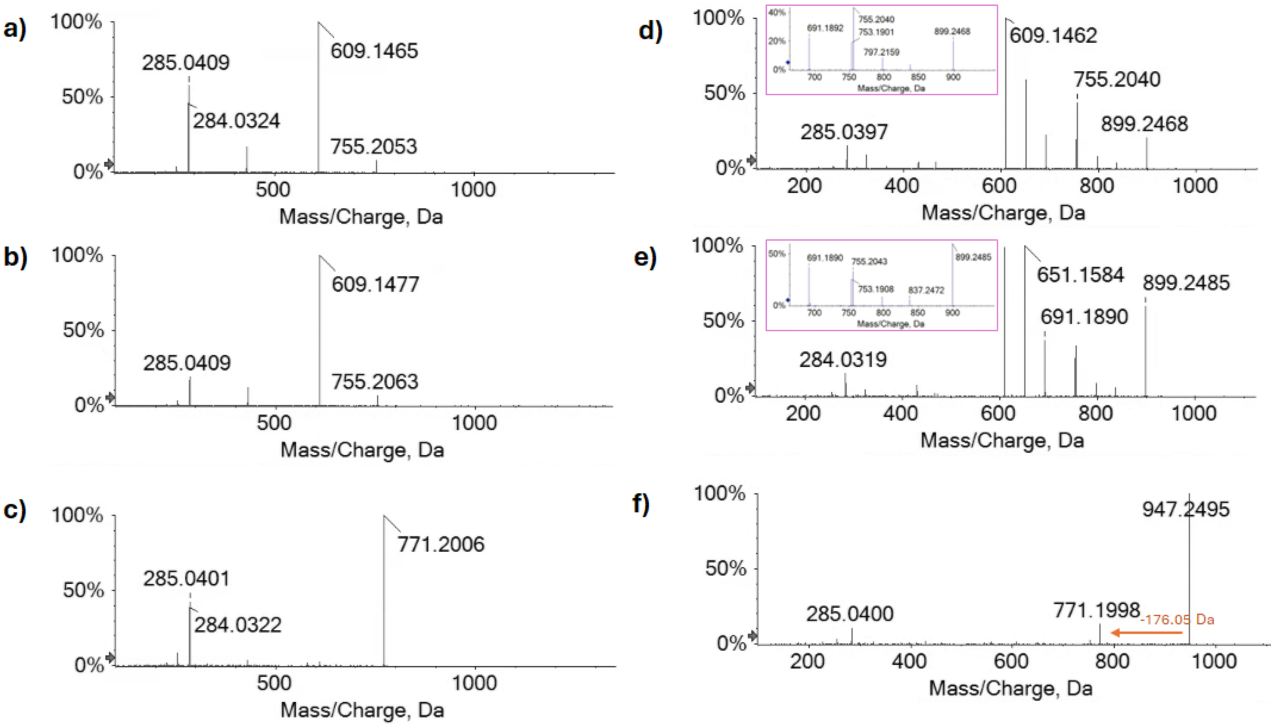

Figure S9. TOF-MS/MS spectra of kaempferol triglycosides **3** (a), **6** (b), **12** (c); the hydroxymethylglutaryl derivatives **11** (d), and **14** (e), and kaempferol feruloyl trihexoside **30** (f).

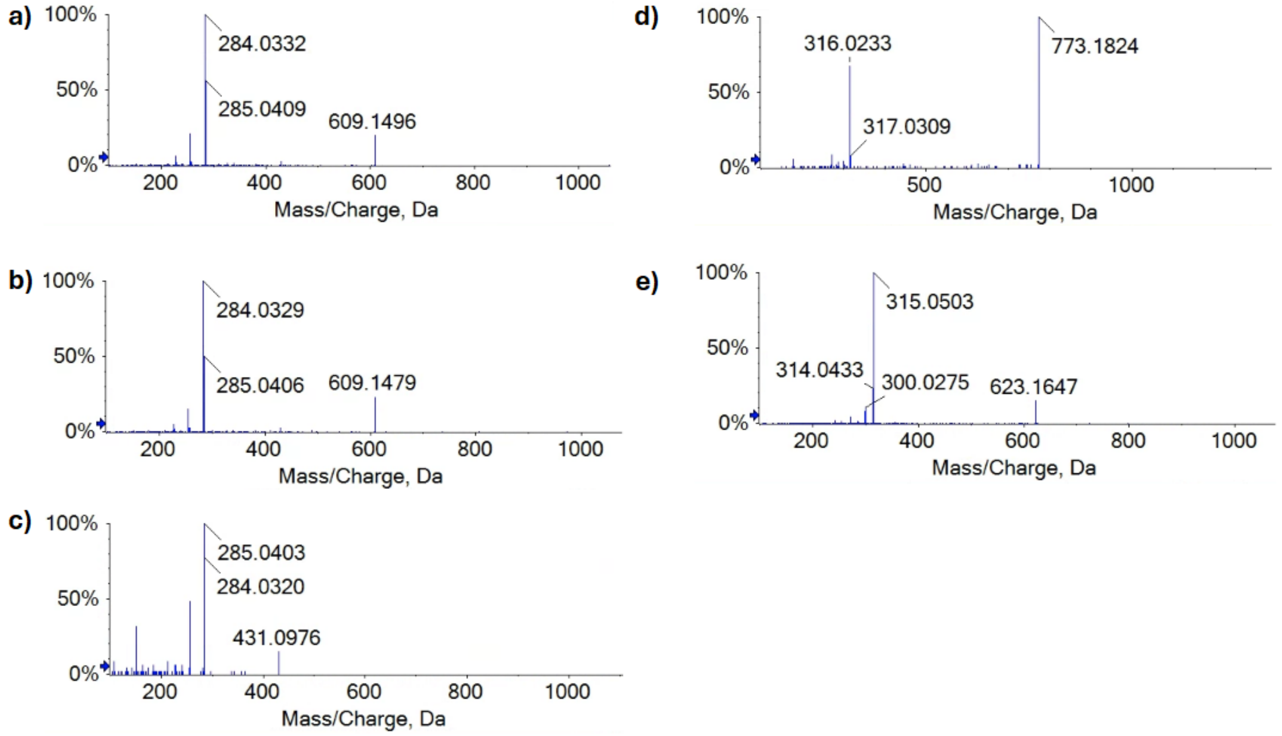

Figure S10. TOF-MS/MS spectra of kaempferol glycosides **4** (a), **17** (b), and **32** (c), and of myricetin triglycoside (**5**) (d) and isorhamnetin dihexoside (**29**) (e).

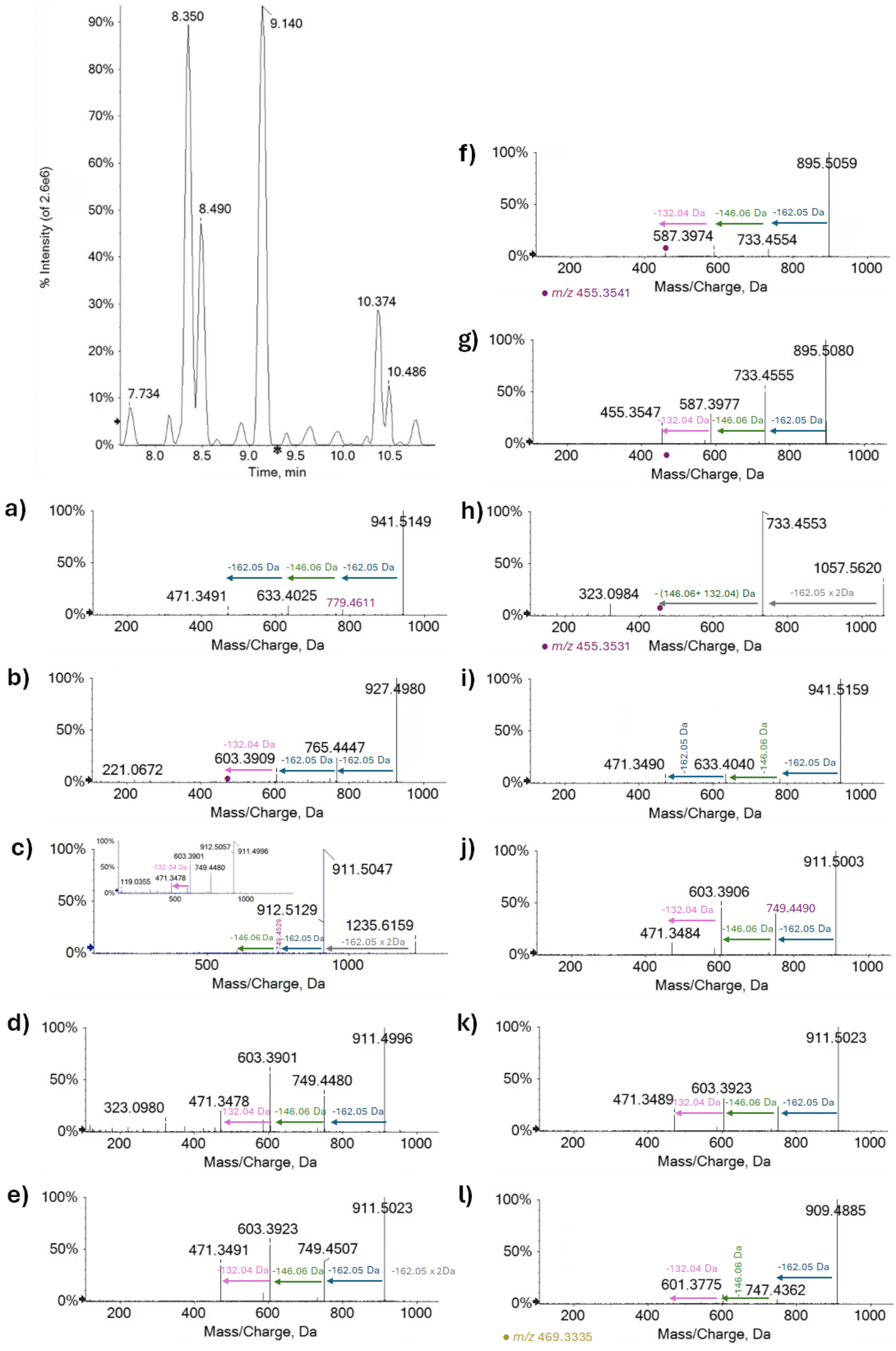

Figure S11. TOF-MS/MS spectra of triterpene saponins **1' – 11'** (a-l). The TIC (Total Ion Chromatogram) region in which the compounds eluted, is shown in the upper part of the figure.

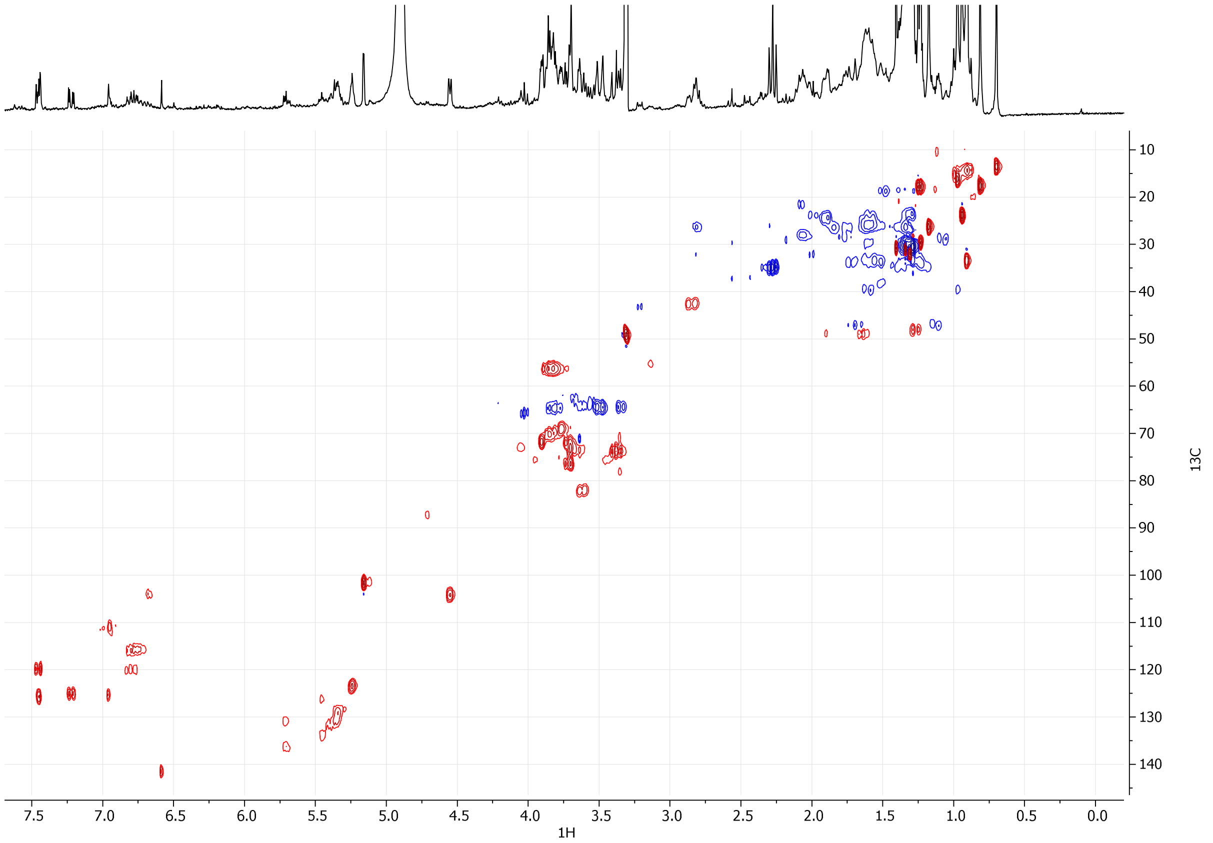

Figure S12. HSQC spectrum of the ethyl acetate fraction obtained from the L/L partitioning of the crude polar extract.

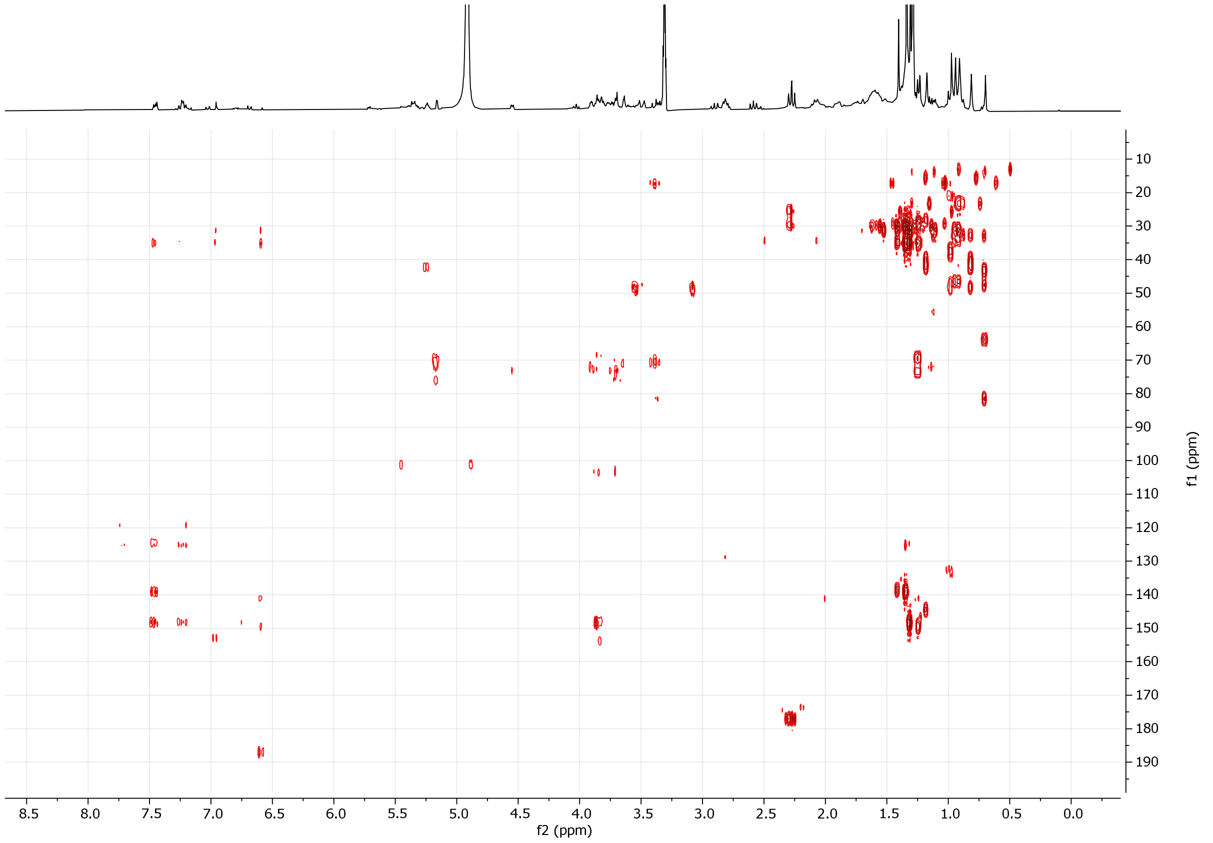

Figure S13. HMBC spectrum of the ethyl acetate fraction obtained from the L/L partitioning of the crude polar extract.
